# Supplementary material for: Confocal Laser Scanning Microscopy of Light-Independent ROS in Arabidopsis thaliana (L.) Heynh. TROL-FNR Mutants
Source: Int J Mol Sci. 2025 Jul 21;26(14):7000. doi: 10.3390/ijms26147000 (PMC12295754; doi:10.3390/ijms26147000)
Supplement: Supplementary file 1 [file ijms-26-07000-s001.zip › ijms-3754836-supplementary.pdf]

Table S1: Physiochemical characteristics of growing substrate under a) normal moisture, b) drought stress

a)

Parameter	Method	Unit	Value determined by analysis
H_2O (105℃)	gravimetry	%	65,52
Dry matter(105℃)	gravimetry	%	34,48
pH (1:2 vol)	electrometry	-	5,64
Electric conductivity (1:2 vol)	conductometry	ms/cm	0,593
Salt	calculation	%	0,076
Organic matter	HRN EN 13039:2012	%	89,03
NH_4-N (1:2 vol)	Bremner method	mg/L	20,84
NO_3-N (1:2 vol)	Bremner method	mg/L	13,42
N-min.	calculation	mg/l	34,31
P_2O_5 (1:2 vol)	spectrophotometry	mg/L	28,74
K_2O (1:2 vol)	flame photometry	mg/L	54,84
Mg (1:2 vol)	AAS	mg/L	5,31
Specific weight	HRN EN 13040:2008	g/cm^3	0,27

b)

Parameter	Method	Unit	Value determined by analysis
H_2O (105℃)	gravimetry	%	42,3
Dry matter(105℃)	gravimetry	%	57,7
pH (1:2 vol)	electrometry	-	6,16
Electric conductivity (1:2 vol)	conductometry	ms/cm	0,029
Salt	calculation	%	0,23
Organic matter	HRN EN 13039:2012	%	90,08
NH_4-N (1:2 vol)	Bremner method	mg/L	2,66
NO_3-N (1:2 vol)	Bremner method	mg/L	1,84
N-min.	calculation	mg/l	4,5
P_2O_5 (1:2 vol)	spectrophotometry	mg/L	12,15
K_2O (1:2 vol)	flame photometry	mg/L	24,73
Mg (1:2 vol)	AAS	mg/L	3,42
Specific weight	HRN EN 13040:2008	g/cm^3	0,309
